# Characterization of antibiotic resistant and enzyme producing bacterial strains isolated from the Arabian Sea

**DOI:** 10.1007/s13205-015-0332-3

**Published:** 2016-01-11

**Authors:** Preeti N. Tallur, Dayanand B. Sajjan, Sikandar I. Mulla, Manjunatha P. Talwar, A. Pragasam, Vinayak M. Nayak, Harichandra Z. Ninnekar, Shivanand S. Bhat

**Affiliations:** 1 Government Arts and Science College, Karwar, Karnataka India; 2 Department of Biochemistry, Karnatak University, Dharwad, Karnataka India

**Keywords:** Extracellular enzymes, *Lysinibacillus sphaericus*, *Vibrio vulnificus*, *Bacillus toyonensis*, Antibiotics, Aromatic compounds

## Abstract

Marine bacteria are known to produce many bioactive molecules and extracellular enzymes of commercial importance. We have investigated the bacterial diversity of the coastal area of Karwar, Karnataka State, India. Among these bacterial isolates, five bacterial strains were selected and identified by their morphological, biochemical characteristics and phylogenetic analysis based on 16S rRNA gene sequences. The identified bacterial isolates, *Bacillus toyonensis* PNTB1, *Lysinibacillus sphaericus* PTB, *Vibrio vulnificus* PMD, *Shewanella* MPTDBS, and *Pseudomonas chlororaphis* PNTB were characterized for their tolerance to salt and antibiotics. *Vibrio vulnificus* PMD showed maximum tolerance at higher concentration of salt than other bacteria. These bacterial strains were screened for the production of extracellular enzymes such as lipase, cellulase, pectinase, tannase, chitinase, and l-glutaminase. *Vibrio vulnificus* showed maximum production of l-glutaminase enzyme. *Bacillus toyonensis* PNTB1 shows lipase, CM-cellulase and chitinase activities. These isolated bacterial cultures were also utilized most of the aromatic compounds at 7 mM. These findings indicate the organisms present in this zone may have more potential applications in bioremediation, agricultural, industrial, and therapeutics.

**Electronic supplementary material:**

The online version of this article (doi:10.1007/s13205-015-0332-3) contains supplementary material, which is available to authorized users.

## Introduction

Marine environment is a source for the isolation of a novel microorganism(s) with the potentiality to produce active secondary metabolites. Among marine organisms, bacteria are of particular interest since they play a vital role in the cycle of matter in water (Rheinheimer 1980). Marine bacteria are well known for their varied bioactive properties, which include the production of secondary metabolites, highly thermostable enzymes and bioactive compounds (Ashadevi et al. 2008). So, it is important to determine the types of bacteria present in the marine ecosystem, the role they play in the functioning of that ecosystem (Nazia and Nuzhat 2006). Hence, in the present study an attempt has been made to isolate and biochemically characterize some of the important marine bacteria from coastal area of Karwar (Karnataka State, India), with a view to identify the organisms and to study their salt tolerance, maximum tolerable concentration of antibiotics, utilization of various aromatic compounds as a growth substrate and also to evaluate their enzyme production potentials. There are reports on the study of marine bacterial diversity from different coastal area of sea (Chiaki et al. 1985; Bozal et al. 2002; Zamudio-Maya et al. 2008; Bal et al. 2009; Aureen et al. 2010; Aisha and Nuzhat 2011). There are very few reports on microorganisms which were isolated from coastal area of Karwar (Pankaj et al. 2011, 2013; Sanjay Kumar and Nagappa 2011; Shreedevi and Rathod 2011). However, there is not much information available on the bacterial cultures isolated from coastal area of Arabian Sea (Karwar, Karnataka State, India) having the ability of producing various extracellular enzymes with maximum tolerance of different antibiotics and salt concentrations and also utilization of various aromatic compounds. Karwar is the coastal city of Arabian Sea and Head Quarters of Uttar Kannada District and is in the West-coast of India, is situated 13^1^55^11^N latitude and 7.40^1^5–75^1^05^1^E longitude. This place is gained a prominent place in the map of India because of the location of the recently commissioned Indian Naval Base called ‘Sea Bird’ and Kaiga Atomic Power Station in its vicinity. In the present study, we describe the cultural, morphological and biochemical characterization of pure bacterial cultures, isolated from marine sediments, west coastal area of Arabian Sea. Further, they were identified on the basis of 16S rRNA gene sequence analysis. In addition, we also present results of the bacterial cultures having the ability to various extracellular enzyme activities with their maximum tolerance towards various antibiotics and salt concentrations. These pure bacterial cultures were also utilizing various aromatic compounds as a growth substrate.

## Materials and methods

### Chemicals

Chlorobenzene, nitrobenzene, phenol, *m*-cresol, *p*-cresol, chitin, l-glutamine, nutrient agar, pectin, tetracycline, streptomycin, ampicillin, ciprofloxacin, penicillin, gentamicin, chloramphenicol, *p*-chloroaniline, *p*-nitrophenol, and penicillin were purchased from Merck, Himedia, SD Fine Chemicals Ltd., Sisco Research Laboratories Pvt. Ltd. (SRL) and Sigma-Aldrich, India. Nutrient media was procured from Himedia (India). All chemicals used are Analytical Reagent (AR) grade.

### Isolation and identification of pure bacterial strains

Samples were taken for serial dilutions up to 10^8^; 100 µl of samples of each dilution were inoculated on the nutrient agar plates for the isolation of bacteria by spread plate technique. The plates were incubated at 37 °C for 48 h, and well separated bacterial colonies with different color were selected for further isolation and purification process. The process was repeated several times to get a pure individual bacterial culture. The pure bacterial cultures were maintained on the nutrient agar slant at 4 °C, sub-culturing once in a month.

The cultural, morphological, and biochemical characteristics of the isolated bacterial strains were studied by adopting the methods described in the standard manuals (Coon et al. 1957; Holding and Collee 1971; Seeley and Van Dan Demark 1972). Gram reaction and morphology of the bacterium were determined from both nutrient agar. The motility test was performed by hanging drop method, and Gram staining was done with ammonium oxalate-crystal violet (Seeley and Van Dan Demark 1972). Spores were observed by staining with malachite green. The pigmentation was observed on King A and King B media. Acid production from sugars and oxidative-fermentation was performed using the method of Hugh-Leifson (1953). Reduction of nitrate and nitrite were tested in liquid medium supplemented with either 0.1 % KNO_3_ or KNO_2_. Nitrate reduction to nitrite was detected by adding naphthylamine and sulphanilic acid reagents as described by Smibert and Krieg (1981). Starch hydrolysis was detected in agar medium supplemented with 1 % soluble starch using iodine indicator. Growth at different temperatures (4–50 °C) and tolerance to different pH (4–12) was determined.

### Identification of bacterial isolate by 16S rRNA gene sequencing

**Fig. 1:**
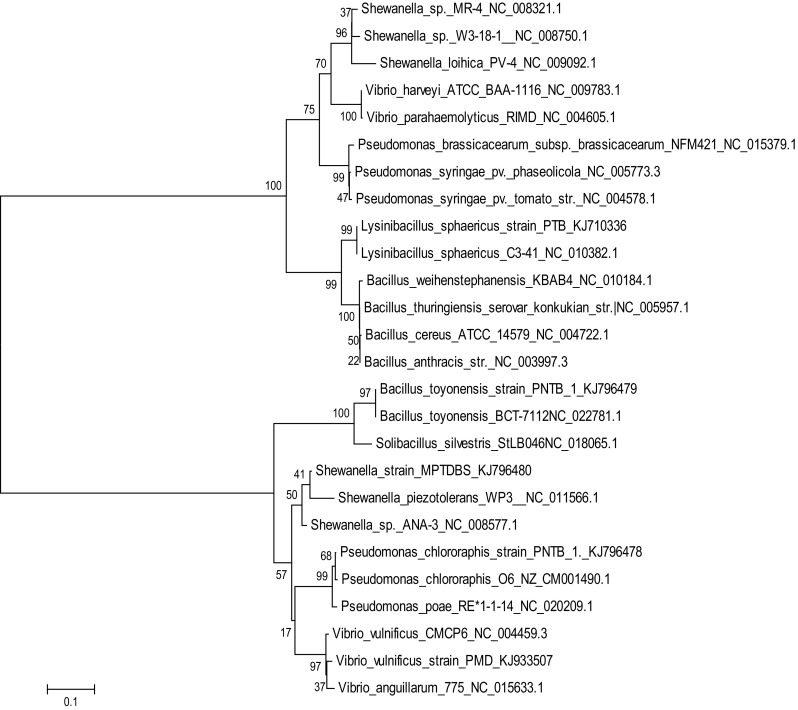
Phylogenetic tree of pure bacterial isolates

The bacterial 16S rRNA gene was amplified from the total genomic DNA using universal specific primers 63F (5′CAGGCCTAACACATGCAAGTC 3′) and 1387R (5′GGGCGGAGTGTACAAGGC 3′), which yielded a product of approximately 1300 base pairs. The polymerase chain reaction (PCR) conditions were 33 cycles of 95 °C denaturation for 1 min, annealing at 55 °C for 43 s and extension at 72 °C for 1 min and in addition one cycle of extension at 72 °C for 7 min. The PCR product was purified by PEG-NaCl precipitation (Sambrook et al. 1989). Briefly, the PCR product was mixed with 0.6 volumes of PEG-NaCl solution (20 % PEG 6000, 2.5 M NaCl) and incubated for 10 min at 37 °C. The precipitate was collected by centrifugation at 12,000×*g* for 10 min. The pellet was washed twice with 70 % ethanol and dried under vacuum, which was resuspended in distilled water at a concentration of >0.1 pmol/ml. The purified product was directly sequenced using a Big Dye Terminator kit (Applied Biosystems, Foster City, USA). The sequencing reactions were run on AB1-PR1SM automated sequencer (ABI-373xl genetic analyzer). The nucleotide sequence analysis was done at the Blastn site at the NCBI server (http://www.ncbi.nlm.nih.gov/BLAST). The alignment of the sequences were done using CLUSTALW program VI.82 at the European Bioinformatics site (http://www.ebi.ac.uk/clustalw). The sequence was refined manually after cross checking with the raw data to remove ambiguities. The phylogenetic tree was constructed using the aligned sequences by the Neighbor-joining method using Kimura-2-parameter distances in the MEGA beta 5.1 software (Tamura et al. 2011). Phylogenetic relationship was established  by Bootstrap method with its bootstrap replication number 1000 and Kimura 2-parameter model as shown in Fig. 1.

### Sample preparation for atomic force microscopy (AFM)

The pure bacterial culture suspension was centrifuged at 9500×*g* for 10 min at 4 °C, to settle the bacterial pellet. The pellet was washed five times with nano pure water. The suspension was filtered with the help of syringe fitted with glass wool. The final purified pellet was resuspended in nano pure water. The bacterial suspension with optimum cell density was directly applied to the clean glass slide and allowed to dry under laminar flow cabinet for 4 h. The air-dried slides were directly analyzed under Nanosurf easyScan 2 AFM **(**Nanosurf AG, Liestal, Switzerland) system in dynamic force with air mode (Greif et al. 2010).

### Screening for extracellular enzymes

The ability of the pure bacterial cultures to produce lipase enzyme was carried out by growing the cultures on tributyrin agar base plates and observing the zone of clearance due to hydrolysis of tributyrin (Sirisha et al. 2010). Cellulose degrading enzyme activity of the isolated bacteria were identified by culturing on Czapeak-Dox medium supplemented with 1 % carboxymethyl cellulase (CMC) according to the method described by Glina and Khatiel (2011). The tannase was tested by growing the cultures on nutrient agar plates containing tannic acid (2 %) and determining the tannase activity according to the method described by Couri and Farias (1995). The YEP medium was used for isolation of pectinase producing bacteria supplemented with 2 % agar (Janani et al. 2011). The chitin utilization was carried out by bacterial in colloidal chitin-agar medium according to the method described by Hackman (Hackman 1962). The l-glutaminase enzyme producing bacteria were isolated according to the method described by Kiruthika and Saraswathy (2013). The total protein concentration was determined by standard Bradford assay using commercial reagent (Bio-Rad, Hercules, USA) according to the instruction manual (Bradford 1976).

### Maximum tolerable concentration for antibiotics and sodium chloride

Maximum tolerable concentrations for antibiotics of the isolated bacteria to different antibiotics were tested on a nutrient agar plate (Well-diffusion method) (Yilmaz et al. 2006). The antibiotics tested for maximum tolerable concentration were tetracycline (TET), norfloxacin (NOR), streptomycin (STP), ampicillin (Amp), ciprofloxacin (CIP), gentamicin (GEN), chloramphenciol (CHL), and penicillin (PEN). Maximum tolerable concentrations of sodium chloride was carried out by the growth of bacteria in nutrient broth containing 5–30 % salt contractions.

### Utilization of various aromatic compounds by pure bacterial cultures

The ability of the individual bacterial cultures to utilize aromatic compounds as a sole source of carbon and energy was determined by measurement of growth in mineral salts medium (MM 1) containing 7 mM of the compound (Hoskeri et al. 2011; Mulla et al. 2012). The growth was measured turbidometrically at 600 nm.

## Results and discussion

**Fig. 2:**
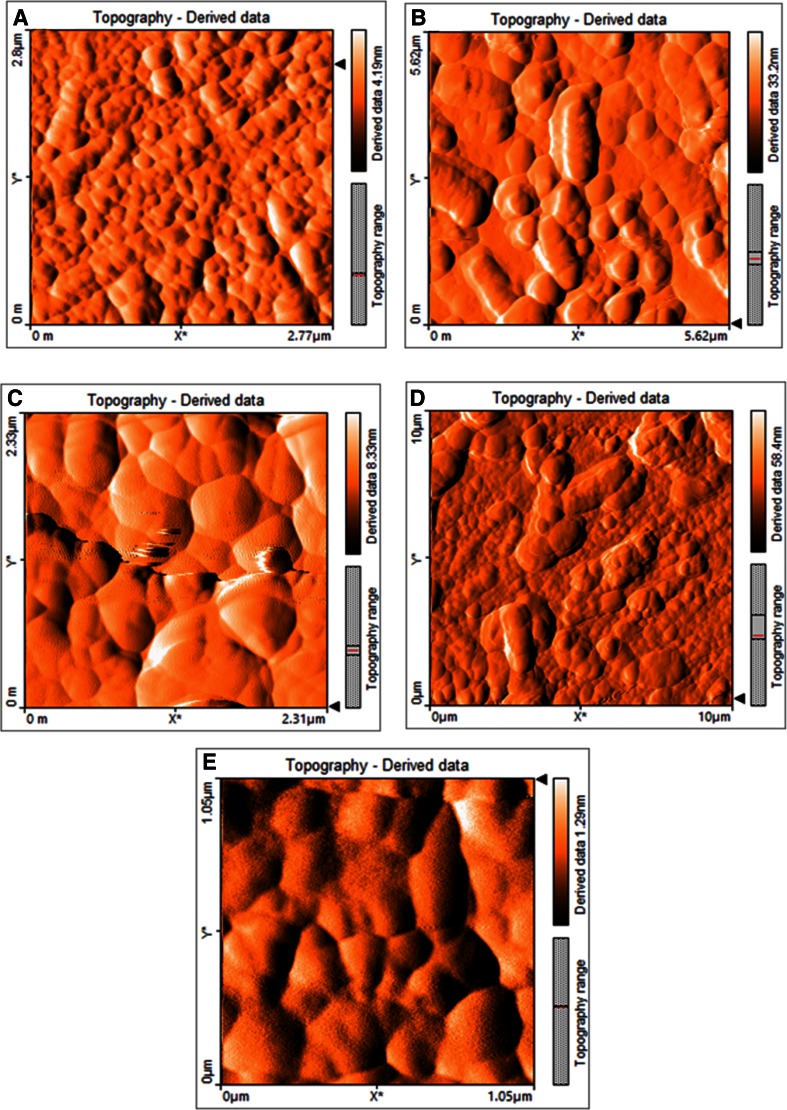
AFM pictures of **a** *Vibrio vulnificus* PMD, **b** *Shewanella* strain MPTDBS, **c** *Lysinibacillus sphaericus* PTB, **d** *Pseudomonas chlororaphis* PNTB, **e** *Bacillus toyonensis* PNTB1

In the present study five commercially exploitable strains of pure bacterial cultures have been selected for extracellular enzyme activities, utilization of various aromatic compounds as a growth substrate, maximum tolerance towards various antibiotics and salt concentrations studies. All the isolated pure bacterial cultures were gram-positive and gram-negative, rod-shaped and aerobic. The cultural, morphological, and biochemical characteristics of the isolated pure bacterial cultures are given in Table 1. According to Bergey’s Manual of Determinative Bacteriology (Holt et al. 1994) and by phylogenetic analysis based on 16S rRNA gene sequences, the isolates were identified as *Bacillus toyonensis* strain PNTB1, *Lysinibacillus sphaericus* strain PTB, *Vibrio vulnificus* strain PMD, *Shewanella* strain MPTDBS, and *Pseudomonas chlororaphis* PNTB. Their sequences were deposited in Genbank under accession number KJ796479, KJ710336, KJ933507, KJ796480, and KJ796478, respectively. The individual pure culture 16S rRNA gene sequence analysis was done at RDP II and NCBI, where relevant sequences from these databases were downloaded for further analysis (Hoskeri et al. 2011). The phylogenetic relationship of isolated individual pure bacterial cultures with other bacterial cultures is shown in Fig. 1. The individual pure bacterial culture is closely aligned with their respective genus between 95 and 100 % (Fig. 1). Also, these pure cultures were analyzed by AFM. AFM is a powerful tool for the analysis of topographical features of bacteria with little or no modification of the sample. It is versatile, robust, fast and economical compared other structural analysis techniques. *Vibrio vulnificus* PMD was found to be rod shaped with an average length of 442.14 nm and height of 23.178 nm (Fig. 2a). *Shewanella* strain MPTDBS was found to be rod shaped with an average length of 1.497 µm and height of 130.54 nm (Fig. 2b). *Lysinibacillus sphaericus* strain PTB has two morphological forms, vegetative rod bacterium, and spherical spores. AFM analysis of spores *L. sphaericus* have typical spherical shapes with an average diameter of 518.5 nm and height of 61.342 nm (Fig. 2c). *Pseudomonas chlororaphis* PNTB1 was found to be rod shaped with an average length of 2.498 µm and height of 284.084 nm (Fig. 2d). *Bacillus toyonensis* strain PNTB1 was found to be rod shaped with an average length of 453.436 nm and height of 9.235 nm (Fig. 2e). Although AFM suffers from minor flaws in the length measurement, due to its probe tip lateral surface interaction with the sample (Kuznetsov and McPherson 2011). Hence, the isolated pure bacterial cultures are the novel strains of their corresponding bacterial genus.

**Table 1:** Cultural, morphological and biochemical characteristics of the isolated pure bacteria

Characteristics	*Bacillus toyonensis* PNTB1	*Lysinibacillus sphaericus* PTB	*Vibrio vulnificus* PMD	*Shewanella* strain MPTDBS	*Pseudomonas chlororaphis* PNTB
Cultural and morphological characteristics					
Colony morphology	Irregular, curled, umbonate, rough, turbid white, opaque	Regular, circular smooth, raised transparent, white	Regular, smooth, circular, concave transparent, pale yellow	Regular, circular, transparent glistening, smooth, yellow	Regular, entire, opaque, smooth, raised, pale yellow
Vegetative cells	Rod	Rod	Rod	Rod	Rod
Motility	+	−	+	+	+
Gram reaction	+	+	−	−	−
Endospores	+	+	−	−	−
Pigment formation	−	−	−	−	Fluorescent diffusible pigment
Temperature (optimum)	10–45 °C (37 °C)	20–40 °C (30 °C)	20–45 °C (30 °C)	15–40 °C (35 °C)	20–40 °C (30 °C)
pH (optimum)	5.5–10 (7.1)	6–9 (7.2)	6–9.5 (7.0)	6.5–9.5 (7.2)	6–9 (7.0)
Biochemical characteristics					
Catalase	+	+	+	+	+
Oxidase	+	+	+	+	+
Urease	−	+	+	+	±
Lysine decarboxylase	+	+	+	+	−
Agrinine dihydrolase	+	–	+	−	−
Starch hydrolysis	+	+	+	−	±
Gelatin hydrolase	+	+	−	+	−
H_2_S production	−	−	−	−	−
Casein hydrolysis	+	−	−	−	−
Indole production	−	−	−	−	−
Ornithine decarboxylase	−	−	+	+	+
Nitrate Reduction	+	+	+	+	+
ONPG	−	−	+	−	−
Citrate utilization	+	−	+	+	−
Methyl red test	−	−	−	−	−
Voges Proskauer test	+	±	−	+	−
Oxidation/fermentation (O/F)					
Mannitol	−	−	O and F	−	−
Glucose	O and F	O and F	O and F	−	O and F
Lactose	−	−	O and F	−	−
Acid and gas production from carbohydrates					
Mannitol	−/−	−/−	±	−/−	−/−
Glucose	±	+/+	±	−/−	±
Sucrose	±	±/−	±	−/−	±
Lactose	−/−	±/−	±	−/−	−/−
Fructose	±	−/−	±	−/−	±
Galactose	±	−/−	±	±	−/−
Maltose	±	−/−	±	−/−	±
Xylose	−/−	−/−	±	−/−	−/−
Arabinose	−/−	±	−/−	−/−	−/−

Cultural and morphological characteristics: + present, − absent; Biochemical characteristics: ± present or absent, − no O/F, ± acid production from carbohydrate/no gas production from carbohydrate, −/− no acid/no gas production, +/+ acid/gas production, ±/− acid or no acid production from carbohydrate/no gas production from carbohydrate

*Lysinibacillus sphaericus* is a spore-forming bacterium used in the biological control of mosquitoes and in bioremediation (Lozano and Jenny 2013; Silva-Filha et al. 2014; Wirth et al. 2014). *Lysinibacillus sphaericus* tolerate and reduces heavy metals and also used in the preparation of nanoparticles (Yujun et al. 2015). Similarly, *Shewanella* strain is also a novel sp., having ability to reduce mercury, iron, uranium, and plutonium metabolically (Ling and Jeremy 2014) and able to synthesize As-S nanotubes via the combined reduction of arsenate and thiosulfate. The biogenic formation of one-dimensional As-S nanotubes greatly contribute to new green biosynthetic methods for the production of inorganic materials at nanoscales, which used in nano and optoelectronic devices (Wiatrowski et al. 2006; Jiang et al. 2009). *Pseudomonas chlororaphis* use as soil inoculants for plants as a bio-controlling agent against *Fusarium oxysporum* fungus, a causative agent of tomato foot and root rot (Chin et al. 2000). *Bacillus toyonensis* is a active ingredient of the preparation of commercial animal nutrient Toyocerin which is used for feeding house hold animals such as swine, poultry, cattle, rabbits and aquaculture (Jiménez et al. 2013).

**Fig. 3:**
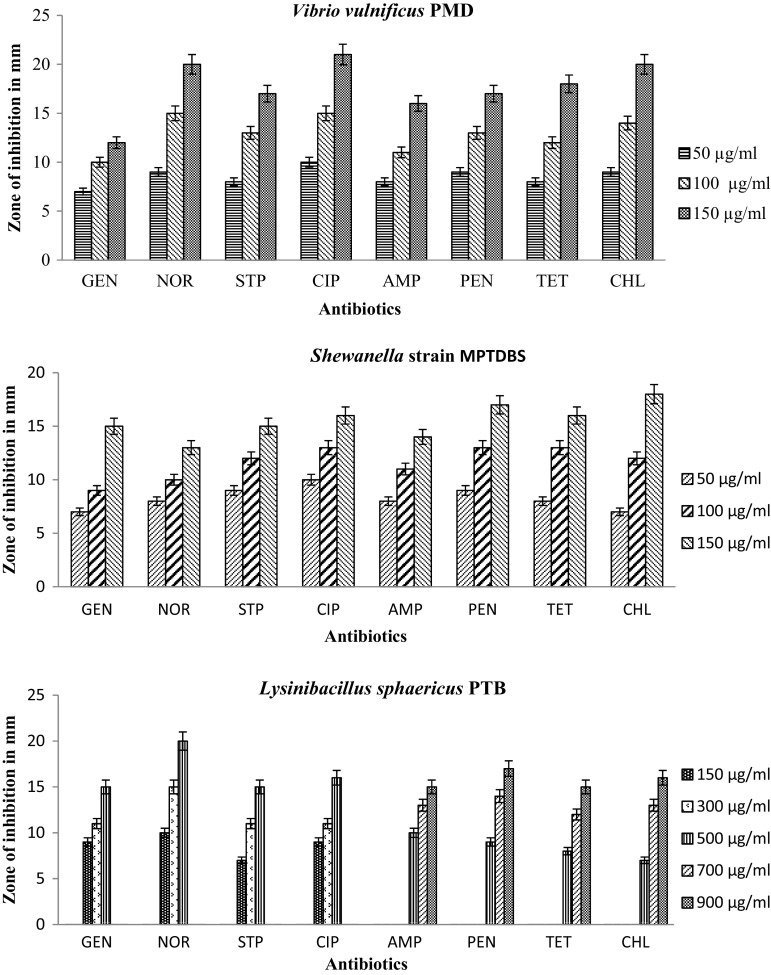

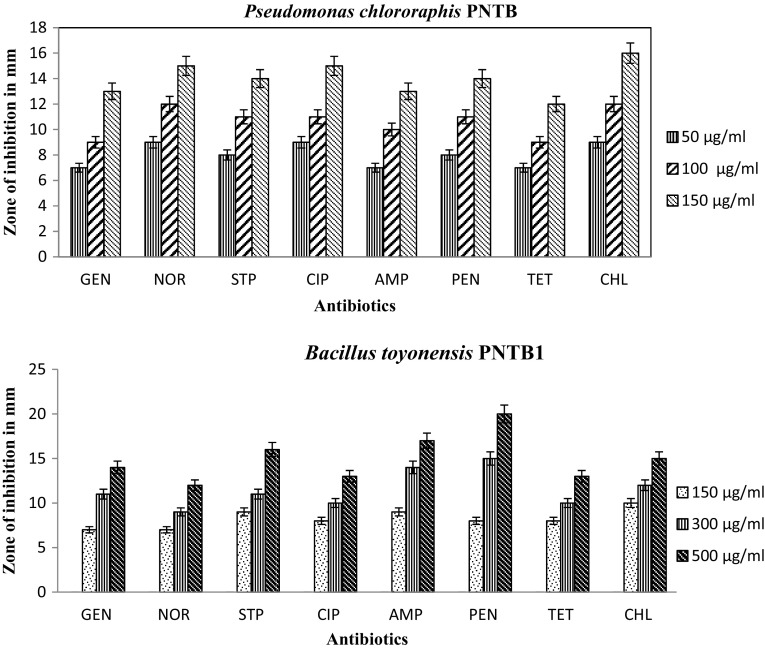
Maximum tolerable concentration for antibiotics of the isolated bacteria

The isolates *Bacillus toyonensis* strain PNTB1, *Lysinibacillus sphaericus* strain PTB shows resistant to lower concentration of antibiotics, *Vibrio vulnificus* PMD, *Shewanella* strain MPTDBS and *Pseudomonas chlororaphis* PNTB were sensitive to lower concentration of antibiotics shown in Fig. 3. The *Vibrio vulnificus* PMD tolerate high concentration of salt compare to other isolates shown in Table 2. A total of 54 isolates were taken for the study of extracellular enzyme activities, out of them *Bacillus toyonensis* strain PNTB1, *Lysinibacillus sphaericus* strain PTB, *Vibrio vulnificus* PMD, *Shewanella* strain MPTDBS, and *Pseudomonas chlororaphis* PNTB were found to be potential source of commercially important enzymes and the activities were found to be maximum in these stains compared to other microorganisms. The extracellular enzyme activities of the isolated bacteria are shown in Table 3. *Vibrio vulnificus* shows glutaminase activity up to 24 U/ml whereas *Streptomyces* sp. showed upto 18.0 U/ml (Krishnakumar et al. 2011). *Lysinibacillus sphaericus* PTB, *Bacillus toyonensis* PNTB1, *Shewanella* strain MPTDBS, and *Pseudomonas chlororaphsis* PNTB showed lipase activity up to 50.58, 49.28, 48.24, and 48.1 U/ml, respectively, whereas *Staphylococcus* sp., and *Bacillus* sp. showed lipase activity up to 48 and 26.37 U/ml, respectively (Sebdani et al. 2011; Sirisha et al. 2010). *Bacillus toyonensis* PNTB1 showed cellulase activity upto 0.47 U/ml whereas *Cellulomonas* sp. showed upto 0.450 U/ml (Muhammad et al. 2012). *Bacillus toyonensis* PNTB1 showed chitinase activity upto 56.12 U/ml whereas *Aeromonas hydrophila* and *Aeromonas punctata* showed up to 43.08 and 53.22 U/ml, respectively (Saima and Roohi 2013). *Bacillus toyonensis* strain PNTB1 shows lipase, cellulase and chitinase activities whereas other isolated pure cultures were not shown cellulase and chitinase activities. *Shewanella* strain MPTDBS and *Pseudomonas chlororaphis* PNTB showed lipase activity. *Lysinibacillus sphaericus* PTB, shows maximum lipase activity compare to other isolates. *Vibrio vulnificus* PMD shows l-glutaminase enzyme activity. l-Glutaminase is a very important enzyme due to its role as a flavor enhancer and antileukemic agent (Krishnakumar et al. 2011; Yulianti et al. 2012; Kiruthika and Saraswathy 2013). The isolates did not show tannase and pectinase activity. Also, the individual pure cultures were utilized various aromatic compounds as their growth substrates (Table 4). *Bacillus toyonensis* strain PNTB1 has showed good growth on MM 1 containing *p*-chloroaniline, chlorobenzene, and *p*-cresol. However, the bacterium does not utilize *m*-cresol as a growth substrate. Similarly, *Lysinibacillus sphaericus* strain PTB showed growth on *p*-chloroaniline, phenol, deltamethrin, but not on *p*-cresol, *m*-cresol and chlorobenzene. *Vibrio vulnificus* PMD showed growth on *p*-chlorophenol but not on *p*-chloroaniline, *p*-nitrophenol an *m*-cresol. *Shewanella* strain MPTDBS showed good growth on nitrobenzene and phenol but not on *m*-cresol and *p*-chlorophenol. *Pseudomonas chlororaphis* PNTB1 showed growth on *p*-chloroaniline, chlorobenzene and deltamethrin but not on *m*-cresol and *p*-chlorophenol. Further experiments on genomics, proteomics and degradation of specific substrates (aromatic compounds) by individual organisms having good growth are under progress. These results are encouraging as isolated bacterial cultures have novel applications.

**Table 2:** Maximum tolerable concentration of sodium chloride

Salt concentration (%)	*Bacillus toyonensis* PNTB1	*Lysinibacillussphaericus* PTB	*Vibrio vulnificus* PMD	*Shewanella strain* MPTDBS	*Pseudomonas chlororaphis* PNTB
5	+	+	+	+	+
10	+	+	+	+	+
12	+	+	+	+	+
15	+	−	+	+	+
20	−	−	+	−	−
25	−	−	+	−	−
30	−	−	−	−	−

+ growth, − no growth

**Table 3:** Extracellular enzyme activities of pure bacterial isolates

Bacteria	Enzyme activity (units/ml)
Lipase	
*Bacillus toyonensis*	49.28 ± 0.01^a^
*Lysinibacillus sphaericus*	50.58 ± 0.02^a^
*Shewanella strain*	48.24 ± 0.04^a^
*Pseudomonas chlororaphis*	48.1 ± 0.02^a^
Cellulase	
*Bacillus toyonensis*	0.47 ± 0.01^a^
Chitinase	
*Bacillus toyonensis*	56.12 ± 0.03^a^
l-glutaminase	
*Vibrio vulnificus*	24 ± 0.01^a^

^a^Enzyme activities are the mean ± SE of assays from triplicate cell free extracts

**Table 4:** Utilization of various aromatic compounds by pure bacterial isolates

Substrate	Growth				
	*B. toyonensis* strain PNTB1	*L. sphaericus* strain PTB	*V. vulnificus* strain PMD	*Shewanella* strain MPTDBS	*P. chlororaphis* strain PNTB1
*p*-Chloroaniline	++	++	_	+	++
Nitrobenzene	−	+	+	++	+
Chlorobenzene	++	−	+	+	++
*p*-Nitrophenol	+	+	−	+	+
Phenol	+	++	+	++	+
*p*-Cresol	++	−	+	+	+
*m*-Cresol	−	−	−	−	−
p-Chlorophenol	+	+	++	−	−
Deltamethrin	+	++	+	+	++
Cypermethrin	+	+	+	+	+

++ good growth, + moderate growth, − no growth

Isolation of bacteria from the marine environment may provide ample scope to assess their therapeutic, pest controlling, industrial, and bioremediation potential. This work represents an emerging view of the bacterial diversity of West coastal area of Arabian Sea, Karwar (Karnataka State, India). Since, there was not much information available on bacterial diversity from Karwar coast. Bacteria isolated in the present study indicate a promising source of microbial genetic resources to be biotechnologically explored.

## Electronic supplementary material

Below is the link to the electronic supplementary material.

Supplementary material 1 (DOCX 65 kb)
