# Supplementary material for: Characterization of antibiotic resistant and enzyme producing bacterial strains isolated from the Arabian Sea
Source: 3 Biotech. 2016 Jan 11;6(1):28. doi: 10.1007/s13205-015-0332-3 (PMC4711286; doi:10.1007/s13205-015-0332-3)
Supplement: Supplementary file 1 — Supplementary material 1 (DOCX 65 kb) [file 13205_2015_332_MOESM1_ESM.docx]

**Supplementary Information**

**Characterization of extracellular enzyme producing and antibiotic resistant marine bacteria isolated from west coast area of Karwar, India**

Preeti N. Tallur,^1*^ Dayanand B. Sajjan,^2^ Sikandar I. Mulla,^2^ ManjunathaP. Talwar,^2^Pragasam A,^1^Vinayak M.Nayak,^1^Harichandra Z. Ninnekar,^2^ and Shivanand S. Bhat^1^

^1^ Government Arts & Science College, Karwar_,_ Karnataka State, India.

^2^ Department of Biochemistry, Karnatak University, Dharwad, Karnataka State, India.

Corresponding Author*

Dr. Preeti N. Tallur

Department of Chemistry

Government Arts & Science College, Karwar-591301

Karnataka, India.

Email Id: [preetiksh2002@yahoo.co.in](mailto:preetiksh2002@yahoo.co.in)

**Supporting Information: 3 pages, 1figure**

**Materials and methods**

**Sampling method**

Sediment/soil samples (approx. 10 gm) were collected in October 2012, from beach areas of the Karwar coast namely Ladies ( 14º, 48', 17.4" N ; 74º, 06', 15.8" E ), Tagore (14º, 49', 42.4" N; 74º, 07', 28.4" E), Devbhag ( 14º, 50', 43.6" N; 74º, 06', 49.2" E ) and Tilmatti ( 14º, 54', 05.9" N; 74º, 05', 04.9" E ) at the depth of one meter from the surface (Supplementary **Figure S1**).The pH and temperature were 8.6 and 27 ^0^C respectively. The samples were placed in sterilized plastic bottles and immediately transferred to the laboratory and kept in the refrigerator until further study.


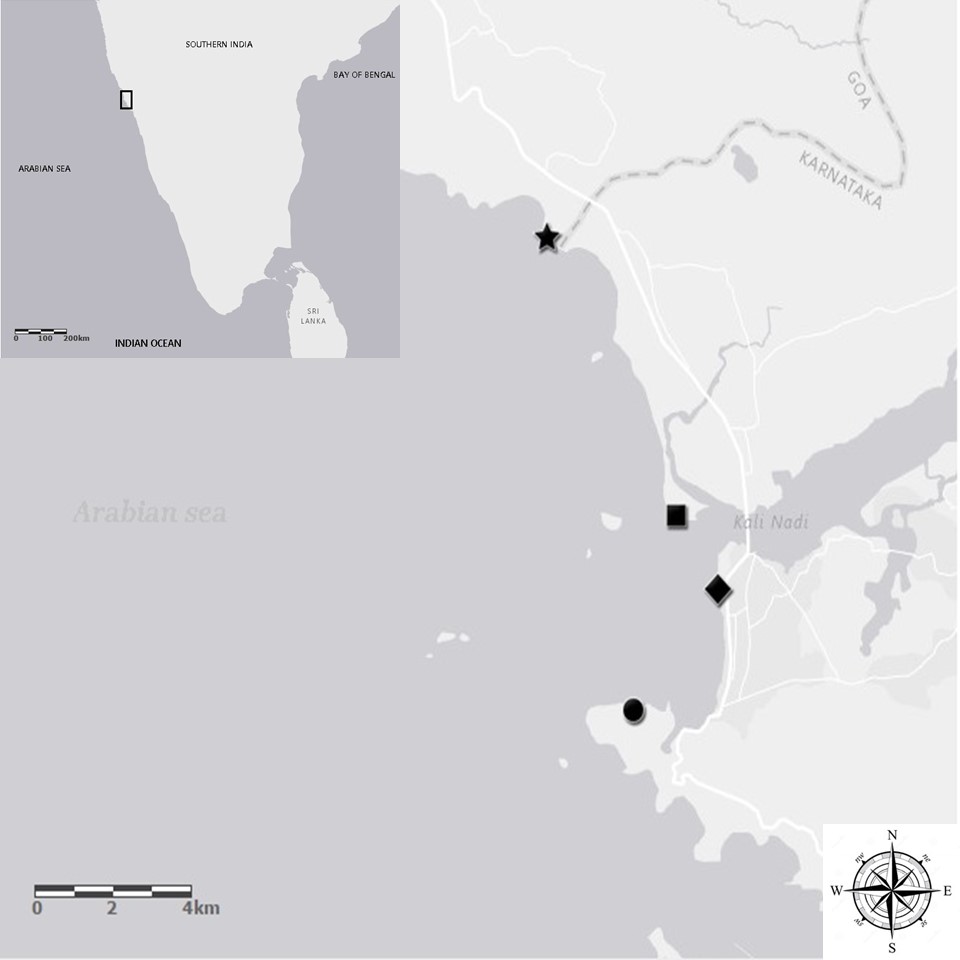


**Figure S1.** The samples were collected around Tilmatti beach (), Devbagh beach (), Tagore beach () and Ladies beach () of South West coast of Karwar (India).
